# Evaluation of insecticide resistance in *Aedes aegypti* populations connected by roads and rivers: the case of Tocantins state in Brazil

**DOI:** 10.1590/0074-02760180318

**Published:** 2019-03-25

**Authors:** Eric Luiz Rodrigues de Sá, Cynara de Melo Rodovalho, Nilciane Pinto Ribeiro de Sousa, Ivy Luizi Rodrigues de Sá, Diogo Fernandes Bellinato, Luciana dos Santos Dias, Luana Carrara da Silva, Ademir Jesus Martins, José Bento Pereira Lima

**Affiliations:** 1 Fundação Oswaldo Cruz-Fiocruz, Instituto Oswaldo Cruz, Laboratório de Fisiologia e Controle de Artrópodes Vetores, Rio de Janeiro, RJ, Brasil; 2 Instituto de Biologia do Exército, Laboratório de Entomologia, Rio de Janeiro, RJ, Brasil; 3 Fundação de Medicina Tropical do Tocantins, Laboratório de Entomologia Médico-Veterinária, Araguaína, TO, Brasil

**Keywords:** resistance monitoring, arboviruses, Amazônia, *kdr* mutation

## Abstract

**BACKGROUND:**

The longstanding application of insecticides for vector control without periodic monitoring of the population response to these chemicals can directly drive the selection of resistant populations of vector mosquitoes. Tocantins is the newest state of the Brazilian Federation. Despite a historically high number of dengue cases, studies and monitoring data concerning insecticide resistance in the state are lacking.

**OBJECTIVES:**

To verify the resistance status of *Aedes aegypti* from 10 populations distributed throughout the state connected by rivers and roads.

**METHODS:**

Between 50 and 150 ovitraps were installed in house gardens within each municipality. Collection points were established based on the importance of the towns and on geographic aspects. Dose response bioassays were performed in accordance with World Health Organization guidelines. Molecular assays were conducted to detect *kdr* mutations, which are related to pyrethroid resistance.

**FINDINGS:**

Of the 3,200 ovitrap paddles analysed, 25.8% contained eggs, with a total of 55,687 eggs collected. With the exception of Caseara, all evaluated populations were considered to be resistant to temephos. The data showed different levels of resistance to deltamethrin among the samples. Caseara and Guaraí showed the lowest RR_95_ values. On average, the Na_V_R1 *kdr* allele was most frequent (40.3%), followed by Na_V_S (38.1%), and Na_V_R2 (21.6%). Palmas, the capital of the state, had the highest frequency of *kdr* alleles (87.5%).

**MAIN CONCLUSIONS:**

With the exception of Palmas, the towns with the highest indexes (ovitrap positivity, number and density of eggs), as well with high levels of resistance and *kdr* alleles were located along the BR-153 road, indicating that the flow of people and cargo can contribute to the dispersion of the vector and potentially resistance. This study contributes substantially to knowledge regarding the insecticide resistance profile of Tocantins mosquito populations; the data generated via the study could facilitate the judicious use of insecticides by vector control programs.

The mosquito *Stegomyia aegypti* (*Aedes aegypti*) (Linnaeus, 1762) (Diptera: Culicidae) is a major vector of dengue, urban yellow fever, chikungunya, and Zika virus. It has a wide distribution in the tropical and subtropical anthropic regions of the world. It utilises for oviposition a large diversity of sites present in the urban environment, particularly discarded manmade materials.^1^

The number of cases involving these arboviruses in Brazil was particularly concerning in 2016. The number of probable cases and deaths was approximately 1,484 million and 701, respectively, for dengue; more than 277 thousand and 216, respectively for chikungunya; and more than 216 thousand probable cases and eight deaths were confirmed for Zika.^2^ In the following year, these numbers decreased, although Brazil continues to be the country with the highest number of dengue cases in the Americas^3^ and has had subsequent new epidemics of yellow fever.^4^

Insecticides have been employed as a method for decreasing the vector density and thereby either resolve an epidemic or reduce it to manageable levels. However, longstanding application, without periodic monitoring of the population response to these chemicals, can directly promote the selection of resistant populations.^5^ This resistance is a result of the selection of genetic alterations that can affect the physiological or behavioural characteristics of insects. These changes can modify insect behaviour, causing it to avoid contact with places treated with insecticides, increase the capacity of the insect to eliminate the compound from the body (metabolic resistance), cause alterations in the binding/active site of the insecticide; or reduce insecticide penetration via cuticle changes.^5^ ^,^ ^6^

*Aedes* resistance to the four major insecticide classes is being detected globally. Resistance to organochlorines, carbamates, but mostly to pyrethroids and organophosphates has been observed worldwide, with some resistance mechanisms well established in distinct populations.^7^ In Brazil, resistance to the larvicide organophosphate temephos and the adulticide pyrethroid deltamethrin is widespread.^8^ ^,^ ^9^ ^,^ ^10^ ^,^ ^11^

The main resistance mechanisms that are selected for relate to the increased activity of detoxifying enzymes and mutations in the molecular target of the insecticide.^6^ Alterations in gene expression rates, as well gene duplications, are recurrent in detox genes, especially those coding for Glutathione-S-transferase (GST), carboxyl-esterases, and multifunctional oxidase P450 super families. Many authors have related pyrethroid resistance in different parts of the world with voltage-gated sodium channel gene (*AaNa*_*V*_ ) mutations [review by Moyes et al.^7^]. Resistance to pyrethroids in Brazilian mosquito populations has been related to reported mutations at the sites 1016 and 1534.^12^

Tocantins is the newest state of the Brazilian Federation, located in the northern region of the country. It was created in 1988 and has 139 municipalities, with an estimated population of 1,550,194 inhabitants in 2017.^13^ Despite the operation of state and municipal services in treating and eliminating breeding sites, the state continues to report an excessive number of dengue cases. Dengue transmission in Tocantins is endemic, with higher incidence between October and May (the rainy season). The circulation of all four virus serotypes was detected in the state, as well the co-circulation of two or more serotypes in many municipalities in previous years.^14^ ^,^ ^15^ Recently, Zika and chikungunya cases have also been reported. This high incidence is exacerbated by the poor economic circumstances and the presence of environmental conditions favourable for mosquito development and arbovirus transmission in almost all municipalities.

The Araguaia, Tocantins, Sono, Balsas, and Paraná rivers, in addition to the many sub-basins, make Tocantins one of the richest states in Brazil with respect to water resources.^16^ The state is divided north to south by the highway Belém-Brasília (BR-153), which has a large flow of cargo and people to various parts of the state and the rest of the country. As shown in the literature, human transportation routes facilitate the dispersal of *Aedes* mosquitoes^17^ ^,^ ^18^ ^,^ ^19^ and diseases, especially if associated with urbanisation and habitat changes.^20^ The towns located along the highway serve as support and overnight dwellings for truck drivers, travellers, and tourists throughout the year. In many towns, the combination of substandard basic sanitation conditions and urban structure (large backyards without walls, a high degree vegetation, and accumulated waste) favour *Ae. aegypti* proliferation and dispersion.

Studies involving arbovirus transmission and vector control are rare in Tocantins state. During the years that the insecticide resistance profile of the Brazilian mosquito populations was evaluated, only two towns in Tocantins (Araguaína and Palmas) were assessed with regard to temephos resistance in separate years (2006 and 2009).^21^ Therefore, to address the lack of data, we aim to investigate the presence and resistance status of *Ae. aegypti* from populations of Tocantins connected by two transfer routes, rivers and roads. We aim to expand the knowledge of the resistance profile of *Ae. aegypti* populations from Tocantins state, identify the mechanisms underlying this resistance, and generate data that can be used by control programs to develop more efficient strategies and operational procedures to combat this vector.

## MATERIALS AND METHODS

**Fig. 1:**
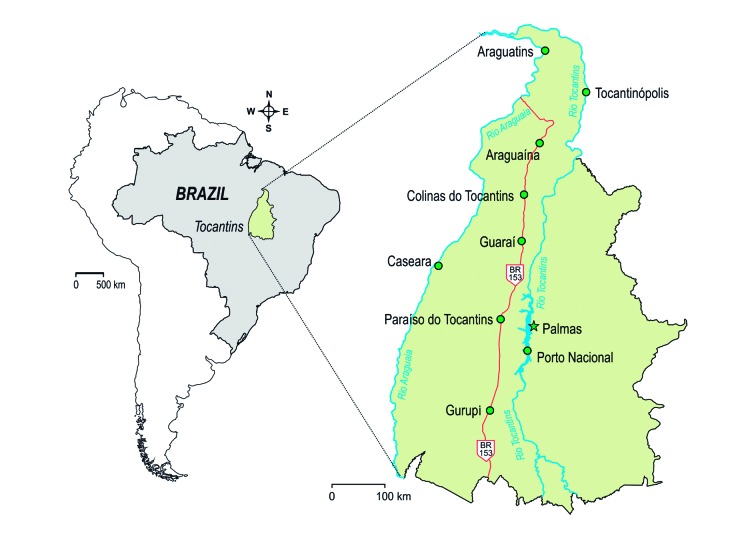
map of Tocantins displaying the municipalities evaluated in the present study. The BR-153 road (red line) and Tocantins and Araguaia rivers (blue lines) are highlighted.

*Study area* - Samples were collected from 10 municipalities. Collection points were established based on the size and economic importance of the towns, geographic distribution throughout the state, and their location along the BR-153 road and the main navigated rivers of Tocantins state, Brazil, shown in Table I and Fig. 1.

**TABLE I:** Sample collection municipalities and their localisation in Tocantins state

Municipality	Localisation	Coordinates
Araguaína	BR-153 road	07° 11’ 27” S 48° 12’ 25” W
Araguatins	Araguaia River	05° 39’ 03” S 48° 07’ 26” W
Caseara	Araguaia River	09° 16’ 40” S 49° 57’ 21” W
Colinas do Tocantins	BR-153 road	08° 03’ 32” S 48° 28’ 30” W
Guaraí	BR-153 road	08° 50’ 02” S 48° 30’ 36” W
Gurupi	BR-153 road	11° 43’ 48” S 49° 04’ 08” W
Palmas	Tocantins River	10° 11’ 04” S 48° 20’ 01” W
Paraíso do Tocantins	BR-153 road	10° 10’ 33” S 48° 52’ 01” W
Porto Nacional	Tocantins River	10° 42’ 28” S 48° 25’ 01” W
Tocantinópolis	Tocantins River	06° 19’ 44” S 47° 24’ 57” W

*Mosquito collections and laboratory rearing* - Collections were conducted in collaboration with the Laboratório de Entomologia Médico-Veterinária at Fundação de Medicina Tropical do Tocantins (Funtrop), with support from Centro de Controle de Zoonoses (CCZ) in Araguaína, Palmas, Guaraí, Gurupi, and Colinas do Tocantins, and Vigilância Epidemiológica in Araguatins, Tocantinópolis, Paraíso do Tocantins, Porto Nacional, and Caseara.

Eggs were collected using ovitraps as described elsewhere,^22^ between December 2011 to June 2012. Between 50 and 150 ovitraps were installed in the grounds of houses across each area. The number of traps used was based on the number of houses in each municipality. The ovitrap paddles were replaced weekly over four weeks and sent to the Funtrop laboratory where they were analysed for the presence of eggs. The egg positivity (EPI) and egg density (EDI) indexes were determined. Eggs were stimulated to hatch and larvae (F0) were reared to adulthood to produce colonies. Adults were separated by species and only *Ae. aegypti* mosquitoes were tested in bioassays, in accordance to World Health Organization (WHO) guidelines. Generations F1, F2, or F3 were used for the assays. Representative samples of F0 mosquitoes were cryopreserved for genotyping assays.

*Insecticide bioassays* - Bioassays were performed according to WHO guidelines.^23^ ^,^ ^24^ In this study, we used mosquitoes from the Rockefeller lineage as a standard pattern of susceptibility.

*Larvicide* - Dose response (DR) bioassays were performed using the organophosphate temephos (Sigma Aldrich, St. Louis, USA). This insecticide was used by mosquito control programs for more than 30 years in Brazil and resulted in great insecticide pressure on the mosquito population. The distribution of temephos in Tocantins ceased in 2013.^21^ Each bioassay consisted of 25 3^rd^-stage larvae (L3) that were exposed over 24 h to 100 mL of insecticide solution in plastic beakers. Four replicates were used for each of the 11 concentrations tested, ranging from 0.0015 to 0.09 mg/L (depending of the population profile). A control group without insecticide was run in parallel, using only the solvent ethanol. For each population, three bioassays were performed on different dates.

*Adulticide* - The resistance profile of adult mosquitoes was evaluated using a DR bioassay with the WHO tube methodology using paper impregnated with insecticide conducted at our Laficave/IOC/Fiocruz facility. The pyrethroid deltamethrin (Sigma Aldrich, St. Louis, USA) was used in 10 different concentrations (varying from 0.5 to 100 mg/m^2^, depending on the population resistance profile), using silicon as the solvent carrier (Dow Corning, Midland, USA). This insecticide was chosen because it is one of the most commonly used adulticides against mosquitoes, both by control programs and in domestic use. Two control tubes, containing only silicone were run in parallel. For each concentration, three repeats of 15-20 non-blood-fed females, 3-to 5-day-old, were exposed to the insecticide tube for 1 h. After exposure, females were gently blown into resting tubes (without insecticide), where a 10% sugar solution was provided for 24 h, after which mortality rates were observed and recorded. For each population the assays were repeated at least three times, on distinct days, under controlled temperature and humidity conditions (26 ± 2ºC and 70 ± 10%, respectively).

For both larvicide and adulticide bioassays, similar procedures were conducted using the insecticide susceptibility reference Rockefeller lineage, provided by Laficave/IOC/Fiocruz.

*Genotyping kdr mutations* - The DNA of 30 males from each population was individually extracted, by maceration in 500 µL of TNES buffer (Tris 50 mM, NaCl 400 mM, EDTA 20 mM, and SDS 0.5%) and incubated with 0.2 mg/L proteinase K in 56ºC for 3 h. DNA was precipitated, alcohol washed and then diluted in 30 µL of TE (Tris 10 mM, EDTA 1 mM) as previously described.^25^ Both 1016 (Val^+^ and Ile^*kdr*^ ) and 1534 (Phe^+^ and Cys^*kdr*^ ) sites of the voltage-gated sodium channel gene (*AaNa*_*V*_ *)* were genotyped using the customised TaqMan Geotyping Assay (Thermo Fischer Scientific, Waltham, USA), with independent reactions for each site, as described by Linss et al.^12^ and Bellinato et al.^11^ DNA from Rockefeller (SS), Rock-kdr [RR, Brito et al.,^26^] and an equimolar mix of Rockefeller and Rock-kdr DNA (RS) were employed as positive controls. Thermocycling conditions followed the manufacturer’s instructions (TaqMan genotyping assay, Thermo Fischer Scientific, Waltham, USA) and the reactions were conducted in real time QuantStudio 6 thermocycler (Thermo Fischer Scientific, Waltham, USA). As previously described,^12^ allelic and genotypic frequencies were determined at sites 1016 and 1534 which are in strong linkage to form a unique locus resulting in six genotypes (SS, SR1, SR2, R1R1, R1R2, and R2R2), composed of three alleles Na_V_S (1016 Val^+^+ 1534 Phe^+^), Na_V_R1 (1016 Val^+^+ 1534 Cys^*kdr*^ ), and Na_V_R2 (1016 Ile^*kdr*^ + 1534 Cys^*kdr*^ ).

*Statistical analysis* - Lethal concentrations (LC) were calculated using Probit analysis, Polo-PC statistics package^27^ and the resistance ratios (RR) were calculated by the division of the field populations LCs and the respective Rockefeller’s lineage LC. Populations were classified according to criteria employed by Mazzari and Georgiou,^28^ in which the populations with RR_95_ < 5 are considered susceptible, RR_95_ between 5 and 10, moderately resistant, and RR_95_ > 10, highly resistant.

Allelic and genotypic *kdr* frequencies were calculated as described elsewhere.^12^ The number of “resistant” genotypes was estimated as the sum of all the homozygous and heterozygous *kdr* genotypes (R1R1, R2R2, and R1R2).

*Ethics* - No ethical licence or permit is required for *Aedes* collection in Brazil. In order to obtain mosquitoes for bioassays, the females were fed using guinea pigs, under the study number L-011/09 approved by the Fiocruz committee (CEUA-Fiocruz).

## RESULTS

From the 800 installed ovitraps, 3,200 paddles were analysed, of these 824 were positive (25.8%) with a total of 55,687 eggs (Table II).

Palmas and Araguaína were the municipalities that had the greatest number of eggs, 22,862, comprising 41% of the total collected in the state. However, the greatest values of EDI were observed in Paraíso do Tocantins (102.9) and Gurupi (83.9).

**TABLE II:** Collection data, paddle positivity, and amount of obtained eggs in each municipality

Municipality	N° traps	N° paddles	Positive paddles	OPI	Total eggs	EDI
Araguaína	150	600	161	26.8	10.045	62.4
Araguatins	50	200	47	23.5	3.513	74.7
Caseara	50	200	11	5.5	417	37.9
Colinas do Tocantins	50	200	127	63.5	6.853	54.0
Guaraí	50	200	91	45.5	5.455	59.9
Gurupi	100	400	47	11.8	3.944	83.9
Palmas	150	600	177	29.5	12.817	72.4
Paraíso do Tocantins	50	200	64	32.0	6.587	102.9
Porto Nacional	100	400	45	11.3	1.890	42.0
Tocantinópolis	50	200	54	27.0	4.166	77.1
Total	800	3.200	824	25.8	55.687	67.6

EDI: egg density index obtained by the ratio between egg number and positive paddles; OPI: ovitrap positivity index, obtained by the percent of positive paddles.

Colinas do Tocantins had the highest percent of positive paddles (63.5%), followed by Guaraí (45.5%), and Paraíso do Tocantins (32). High numbers of eggs were also collected from these areas, even though only 50 traps were installed in each town due to the low number of properties. The contribution in number of eggs of these three municipalities represented 34% of the total collected in the state. Colinas do Tocantins and Guaraí displayed a similar EDI (54 and 59.9, respectively).

The lowest quantity of eggs, positive paddles, and EDI were registered in Caseara, with only 417 collected eggs, representing 0.7% of the total of all municipalities, 11 positive paddles (5.5% of the 200 evaluated), and EDI of 37.9.

**Fig. 2:**
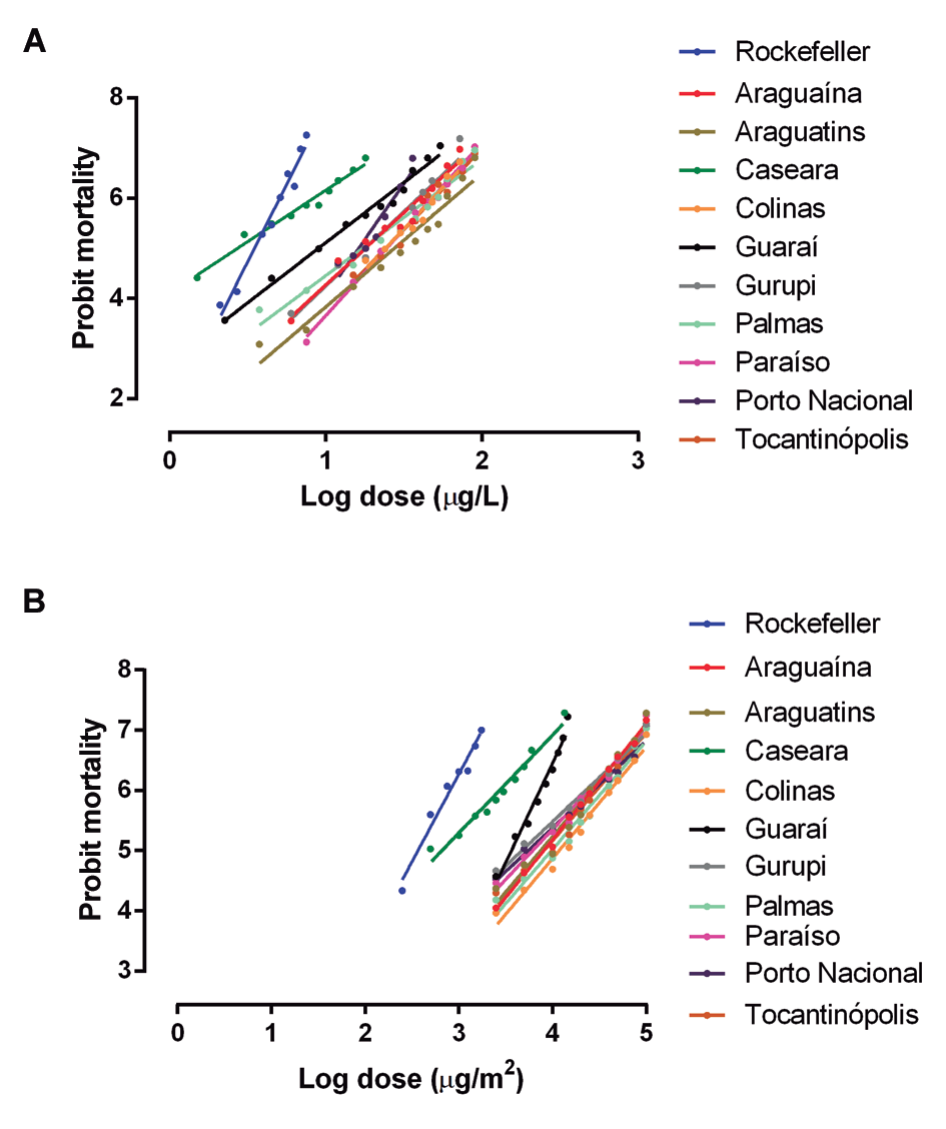
linear regression of *Aedes aegypti* mortality after exposure to the organophosphate temephos (A) and the pyrethroid deltamethrin (B). All populations collected in Tocantins state were evaluated and compared with the control Rockefeller lineage (in blue).

*Bioassays with larvae* - Fig. 2A and Table III summarise the results of DR bioassays performed with the organophosphate temephos, the primary larvicide used for decades against *Ae. aegypti* in Brazil. All the evaluated populations were considered resistant to temephos, with the exception of Caseara. Three populations (Porto Nacional, Gurupi, and Guaraí) showed moderate resistance, while the majority displayed RR_95_ above 10. Comparison of slope values showed that all populations were more heterogeneous than the Rockefeller lineage.

**TABLE III:** Resistance profile of different populations from Tocantins to the larvicide temephos, with lethal concentration (LC) values in mg/L and confidence intervals (CI)

Population	Generation	Dose response					RR	
		LC50	CI50	LC95	CI95	Slope	RR50	RR95
Rockefeller		0.004	0.003-0.004	0.007	0.006-0.007	6.153	1.0	1.0
Araguaína	F1	0.018	0.001-0.019	0.071	0.065-0.078	2.724	4.5	10.1
Araguatins	F1	0.030	0.028-0.031	0.108	0.098-0.118	2.954	7.5	15.4
Caseara	F1-F2	0.003	0.002-0.003	0.018	0.016-0.021	2.000	0.8	2.6
Colinas do TO	F1	0.024	0.023-0.025	0.080	0.073-0.088	3.130	6.0	11.4
Guaraí	F1	0.009	0.008-0.010	0.051	0.041-0.050	2.322	2.3	7.3
Gurupi	F1	0.018	0.017-0.019	0.065	0.060-0.071	2.962	4.5	9.3
Palmas	F1	0.018	0.017-0.019	0.092	0.083-0.102	2.342	4.5	13.1
Paraíso do TO	F1	0.024	0.023-0.025	0.074	0.069-0.080	3.374	6.0	10.6
Porto Nacional	F1	0.016	0.016-0.017	0.040	0.037-0.044	4.184	4.0	5.7
Tocantinópolis	F1	0.024	0.023-0.026	0.079	0.073-0.086	3.218	6.0	11.3

RR: resistance ratio. Populations with RR_95_ < 5 were considered susceptible, RR_95_ between 5 and 10 were considered moderately resistant and RR_95_ > 10 as resistant.

*Bioassays with adults* - DR bioassay results for the mosquito populations exposed to the pyrethroid deltamethrin, indicated that all evaluated populations were resistant to this insecticide. The data revealed varying levels of resistance between the samples, with RR_95_ ranging from 6 to 74.4 (Fig. 2B, Table IV). Caseara and Guaraí had the lowest RR_95_ values (6 and 9.3, respectively). Values for all other municipalities were above 40 (Table IV). Based on a comparison of the obtained slopes, all populations presented greater heterogeneity than the Rockefeller lineage, except for that from Guaraí.

**TABLE IV:** Resistance profile of different populations from Tocantins to the adulticide deltamethrin, with lethal concentration (LC) values in mg/m^2^ and confidence intervals (CI)

Population	Generation	Dose response					RR	
		LC50	CI50	LC95	CI95	Slope	RR50	RR95
Rockefeller		0.369	0.339-0.401	1.288	1.169-1.418	3.029	1.0	1.0
Araguaína	F2	8.083	7.202-9.071	57.969	48.668-69.048	1.922	21.9	45.0
Araguatins	F2	7.545	6.689-8.512	64.566	53.822-77.455	1.764	20.4	50.1
Caseara	F3	0.606	0.487-0.755	7.672	6.008-9.797	1.492	1.6	6.0
Colinas do TO	F3	12.216	10.744-13.891	95.768*	75.415-121.615	1.839	33.1	74.4
Guaraí	F2	3.577	3.265-3.919	11.997	10.768-13.365	3.129	9.7	9.3
Gurupi	F1	4.563	3.749-5.555	66.562	52.067-85.091	1.413	12.3	51.7
Palmas	F2	10.022	8.922-11.257	88.369	72.145-108.242	1.739	27.2	68.6
Paraíso do TO	F2	6.025	5.175-7.015	64.618	52.277-79.874	1.596	16.3	50.2
Porto Nacional	F2	5.370	4.487-6.427	79.392	61.943-101.756	1.406	14.6	61.6
Tocantinópolis	F2	8.204	7.240-9.296	72.949	59.843-88.924	1.733	22.2	56.6

RR: resistance ratio. Populations with RR_95_ between 5 and 10 were considered moderately resistant and RR_95_ > 10 as resistant; *: the assays with this population reached mortality up to 58.7% and, thus, the LC_95_ was obtained by program extrapolation.

**Fig. 3:**
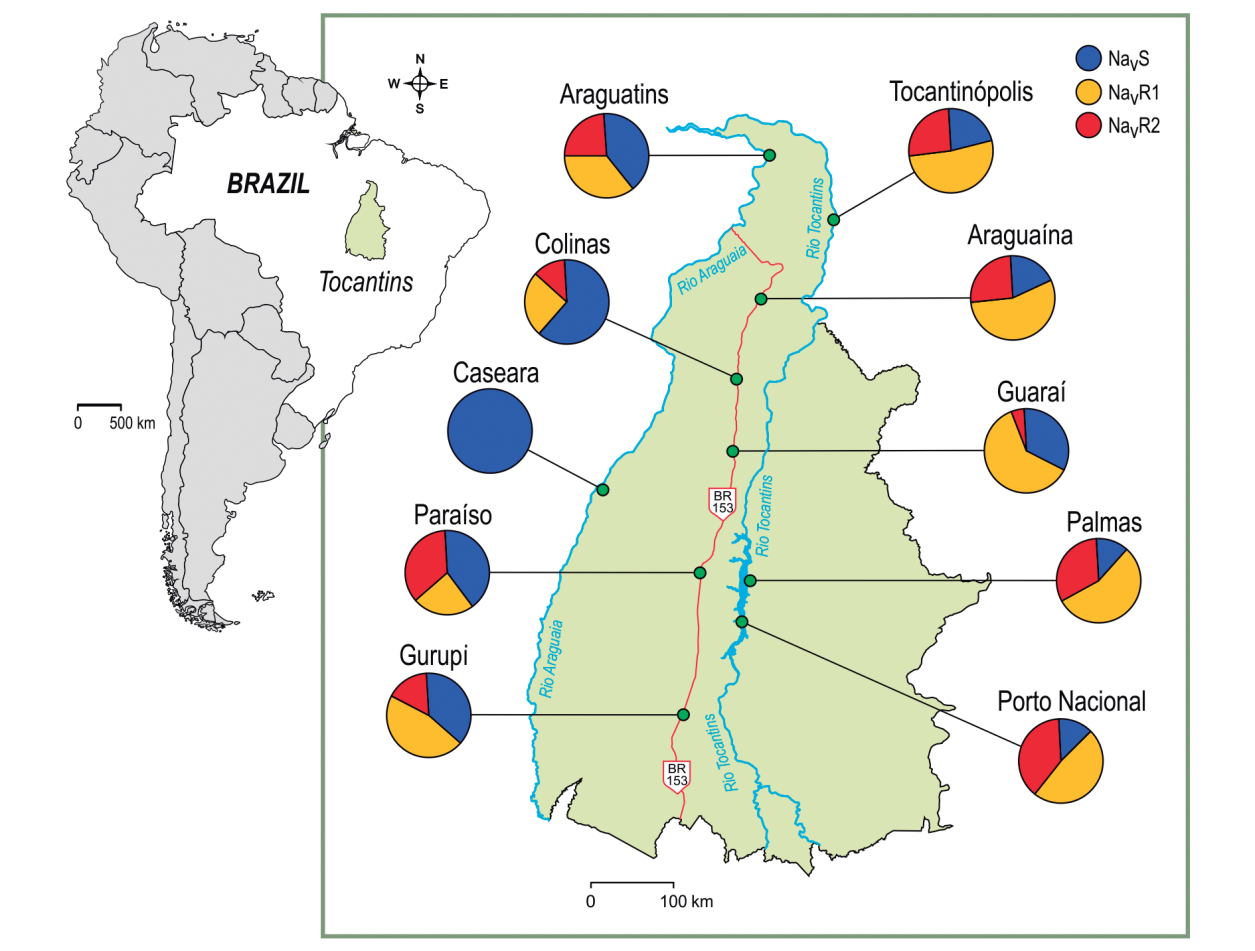
distribution of allelic frequencies of the *Na*_*V*_ sites 1016 and 1534 in *Aedes aegypti* populations from Tocantins, Brazil, considering the alleles Na_V_S (1016 Val^+^+ 1534 Phe^+^), Na_V_R1 (1016 Val^+^+ 1534 Cys^*kdr*^ ), and Na_V_R2 (1016 Ile^*kdr*^ + 1534 Cys^*kdr*^ ).

*Molecular assays to kdr mutation diagnostic* - Molecular assays for the detection of two mutations related with pyrethroid resistance (*kdr* mutations) were conducted on all 10 study populations. The sites 1016 and 1534 in *AaNa*_*V*_ segments IIS6 and IIIS6, respectively*,* were individually genotyped and the results analysed for both sites in linkage and as an individual *locus*.^12^ Fig. 3 and Table V show allelic and genotypic frequencies, respectively, for *Ae. aegypti* populations from Tocantins. Of note, Na_V_R1 (1016 Val^+^+ 1534 Cys^*kdr*^ ) and Na_V_R2 (1016 Ile^*kdr*^ + 1534 Cys^*kdr*^ ) *kdr* alleles were present in all investigated populations, except for Caseara, where only the wildtype allele Na_V_S (1016 Val^+^+ 1534 Phe^+^) was observed.

On average, the Na_V_R1 *kdr* allele was most frequent (40.3%), followed by Na_V_S (38.1%) and Na_V_R2 (21.6%). The locality with the highest frequency of *kdr* alleles was Palmas (87.5%), the state capital, whereas the highest frequency of Na_V_R1 was found in Guaraí (61.7%), and Porto Nacional was the municipality with the highest frequency of Na_V_R2 (38.3%) (Fig. 3).

With relation to genotypes (Table V), Palmas and Porto Nacional showed greater chances of having pyrethroid resistance via the mechanism of target site alteration, with 75% and 73.3% of individuals, respectively, presenting a resistant genotype (R1R1, R1R2, or R2R2). The lowest frequencies for resistant genotypes were found in Caseara (0%), Colinas do Tocantins (17.9%), and Paraíso do Tocantins (21.4%).

**TABLE V:** Genotypic frequencies of *Aedes aegypti* populations from Tocantins considering the alleles Na_V_S (1016 Val^+^+ 1534 Phe^+^), Na_V_R1 (1016 Val^+^+ 1534 Cys^*kdr*^ ) and Na_V_R2 (1016 Ile^*kdr*^ + 1534 Cys^*kdr*^ )

Population	Total	Genotypic frequencies						“Resistant” genotypes*
	(n)	SS	SR1	R1R1	SR2	R1R2	R2R2	
Araguaína	29	0.034	0.241	0.241	0.069	0.379	0.034	0.655
Araguatins	25	0.240	0.200	0.080	0.120	0.360	0	0.440
Caseara	27	1	0	0	0	0	0	0
Colinas do TO	28	0.429	0.214	0.107	0.179	0.071	0	0.179
Guaraí	30	0.167	0.300	0.433	0.033	0.067	0	0.500
Gurupi	24	0.083	0.458	0.125	0.125	0.208	0	0.333
Palmas	28	0	0.071	0.393	0.1786	0.250	0.107	0.750
Paraíso do TO	28	0.036	0.286	0	0.464	0.179	0.036	0.214
Porto Nacional	30	0	0.200	0.267	0.067	0.233	0.233	0.733
Tocantinópolis	23	0.043	0.174	0.304	0.174	0.261	0.043	0.609

*: R1R1 + R1R2 + R2R2.

## DISCUSSION

In this study, mosquito eggs were sampled from municipalities representing different parts of Tocantins state and 10 *Ae. aegypti* populations were investigated for resistance profiles to the insecticides temephos (organophosphate) and deltamethrin (pyrethroid) and for the frequency of *AaNa*_*V*_ mutations related with pyrethroid resistance.

Analysis of the collection data indicated that the towns with the highest indexes (ovitrap positivity and number and density of eggs) were those located along the BR-153 road. This suggests that the flow of people and cargo contributes to the dispersal of the vector. Palmas was an exception to this observation. This city is located by the Tocantins river and was the municipality with the highest number of eggs. Although it is not crossed by the BR-153 road, it is the capital of the state, the biggest town, and in common with towns along the BR-153, has a very high flow of people and cargo.

The adoption of criteria for resistance classification and data analysis is necessary for system standardisation to allow comparison between various studies. At present, definitions are often arbitrary, and many studies do not define the specifics of each assay, the mode of action of the insecticide, and the impact of resistance in the field.

For DR assays, the WHO guidelines recommend the use of the following criteria: the population is considered susceptible at RR_50_ < 5, moderately resistant at RR_50_ between 5 and 10, and highly resistant at RR_50_ > 10.^29^ The use of RR_50_ is recommended because in this range there are smaller confidence intervals and more reproducibility.

Mazzari and Georgiou^28^ employed similar criteria but used RR_95_ rather than RR_50_ as recommended by the WHO. However, the authors did not discuss their chosen values and did not explain their rationale for adopting such criteria.

In Brazil, the National Program of Dengue Control (Programa Nacional de Controle de Dengue - PNCD) employed operational criterion to evaluate temephos resistance, which was expanded later to other insecticides. According to this criterion, populations with RR_95_ > 3 were considered resistant. The use of this threshold allows for the time that the Ministry of Health, as well as municipalities, take to replace the insecticides used in the control programs. Owing to this delay, resistance could continue to increase until the insecticides used are replaced and the PNCD is attempting to find a way to preserve the product such that it is suitable for future use, before the level of resistance becomes too high in the field.

In this study, we followed the criteria employed by Mazzari and Georgiou^28^ because we believe that heterogeneous populations show intermediate LCs that can vary vastly due to the presence of both more and less resistant individuals, thus, the obtained values of RR_50_ may not reflect the true profile of the population. Moreover, the use of RR_95_ is preferable for comparison of different populations, because the LCs used effectively kill all individuals, including those most resistant.

Of the 10 evaluated populations, nine showed resistance to temephos, while the population from Caseara did not. The lowest resistance ratios were found in municipalities by the Araguaia (Caseara) and Tocantins (Porto Nacional) rivers, which are further away from the federal highway that crosses the state. Caseara is also less urbanised and a significant distance from other large urban centres. Again, this indicates that greater proximity to urban centres and consequently, a greater flow of people, can contribute to *Ae. aegypti* infestation, and potentially, to the spread of resistance.

Access to the Caseara municipality from the capital, Palmas, is via a state highway (TO-080) that crosses a typical cerrado (savannah) area of more than 260 km, and where there are only three small cities separated from one another by great distances. This route passes through a few villages and many cattle and soy farms. Caseara can also be accessed via São Domingos municipality, a part of Mato Grosso state, that is located 1 km away on the other side of the Araguaia river. Transport between the cities consists of small boats or car ferries. Due to the difficulty in access, *Aedes* infestation occurred later at this location and, of the municipalities evaluated, this was the last to register the presence of these mosquitoes. Moreover, few dengue cases have been reported here over the years. As a consequence, larval control of *Ae. aegypti* at this site is much more recent, and the local mosquito population has therefore not been subjected to as high selection pressure from the larvicide as have other municipalities.

Unlike Caseara, the remaining municipalities were priority to PNCD (http://portalarquivos2.saude.gov.br/images/pdf/2017/maio/03/001-TO-Relatorio-de-Situacao.pdf). Vector control actions were therefore more intensive, including the use of larvicides; this explains the level of resistance we observed in our bioassay results.

Resistance to temephos is widespread in other regions of Brazil.^11^ The dynamics of resistance acquisition can be linked to many factors, including the history of insecticide use, the level of pre-existing susceptibility to the compound, the frequency and heritability of genes linked to resistance, and the co-selection of distinct resistance mechanisms in the same population.^30^ Although the history of temephos use in the studied municipalities is not presented here, the evaluated *Ae. aegypti* populations showed evidence of being under selection pressure owing to the treatment of domestic breeding sites with temephos by endemic control services. According to Bellinato,^21^ temephos was distributed in Tocantins state until 2013. Insect growth regulators were distributed to the state in 2004 and later in 2010; this distribution has been continued throughout the years since.

The different levels of resistance found and more importantly, the calculated RR values for the municipalities of Tocantins demonstrated the necessity for frequent monitoring of populations. This will allow control programs to perform effective insecticide management, and preserve the product as well as the environment, with the possibility to reduce resistance levels.

Braga et al.^31^ and Lima et al.^10^ observed that there was a reduction in resistance levels to temephos in Brazilian localities where the use of this insecticide was replaced by biological control with *Bacillus thuringiensis* (Bti). In Juazeiro do Norte, a municipality of Ceará state, a decrease in resistance of 30% was noted between 2003 and 2009 (with RR_95_ changing from 10.4 to 7.4) caused solely by the cessation of the use of temephos. In the same study, Lima et al.^10^ observed that in two other municipalities of the same state, Barbalha and Crato, which continued the use of temephos, resistance levels increased from 7.5 to 30 and 9 to 192.7, respectively.

High resistance levels (greater than 10) may have an impact in the field. Montella et al.^9^ showed low persistence of temephos in simulated field tests performed with populations with RR > 10. In addition, the authors found a decrease in *Ae. aegypti* resistance (RR_95_ in 2004 was 18.6 and reduced to 8.2 in 2007) in a municipality of the metropolitan region of Natal (Rio Grande do Norte state), after the replacement of temephos with *Bti* in 2005.

Examination of temephos resistance in the context of the geographical locations of the municipalities revealed that five (Araguatins, Tocantinópolis, Araguaína, Colinas, and Guaraí) of the nine resistant municipalities were located in the north of the state, three (Palmas, Paraíso do Tocantins, and Porto Nacional) in the central region, and one (Gurupi) in the south, emphasising that resistance spread across the state, with no obvious pattern with respect to the levels of resistance observed. Carvalho et al.^8^ demonstrated that the distance between cities is not related to the susceptibility levels of populations.

The results of DR bioassays revealed that all the evaluated populations in this study were resistant to deltamethrin. Eight of the 10 evaluated populations had high resistance, with RR_95_ ranging from 45 to 74.4. The other two, Guaraí and Caseara, had lower RR_95_ values, 9.3 and 6, respectively.

Resistance of *Ae. aegypti* populations to pyrethroids, including deltamethrin, has been demonstrated in multiple countries across different regions of the world. Moyes et al.^7^ documented resistance in several countries in South America, Central America, and North America; a few African countries; and several Asian and Oceania countries. In Brazil, populations resistant to deltamethrin are found in almost all states and the main mechanism involved is target site alteration, namely *kdr* mutations.^11^ ^,^ ^12^

In this study, we evaluated only one resistance mechanism, target site alteration, via the detection of *kdr* mutations related to pyrethroid resistance. However, it was possible to verify that different mechanisms were selected for temephos and deltamethrin resistance. For example, the population from Araguatins showed the greatest temephos resistance in the bioassays (RR_95_ = 15.4), but was not among the populations with the highest levels of resistance to deltamethrin (RR_95_ = 50.1) and displayed a median frequency of resistance genotypes among the evaluated populations.

The Caseara population showed a frequency of 100% for susceptible alleles (wildtype), that is, the *kdr* mutations that confer pyrethroid resistance were not detected. With this in mind, we can infer that the low resistance to deltamethrin (RR_95_ = 6) found for this population was due to other selected mechanisms and not target site alteration.

Colinas do Tocantins presented interesting observations. The RRs for deltamethrin were the highest found among all evaluated populations (RR_50_ = 33.1 and RR_95_ = 74.4) and these values classify it as extremely resistant. However, the molecular analysis results revealed a frequency of more than 60% of wildtype alleles. This information allows us to infer that the resistance observed may not be completely due to target site alteration, but a result of another selected mechanism, probably metabolic resistance.

The opposite situation was observed for the Guaraí population, which presented a median resistance in the bioassays (RR_50_ = 9.7 and RR_95_ = 9.3) but the molecular analysis showed a frequency of more than 60% of Na_V_R1 and Na_V_R2 alleles.

Observing the number of probable dengue cases registered over the years [Supplementary data (Table)], it is possible to note that the towns with fewer cases, Caseara and Guaraí, had the lowest levels of resistance to temephos and deltamethrin. Conversely, Palmas, the municipality with the highest number of probable cases reported, presented very high levels of resistance to both insecticides, as well the highest frequency of resistant genotypes. With the exception of Guaraí, the municipalities along the BR-153 road reported a great number of dengue cases since 2000 and had high levels of resistance and *kdr* allele frequencies.

The reporting of dengue cases is followed up by vector control program action and in most cases, this involves the use of insecticides. Moreover, the increase in the number of disease cases causes fear among the population, resulting in an increase in the use of domestic insecticides. Therefore, we expect higher levels of resistance in places with a reported history of mosquito presence and arbovirus circulation.^21^

In Tocantins, the economic and environmental conditions are favourable for *Ae. aegypti* development and arbovirus transmission. This is combined with increasing numbers of dengue, Zika, and chikungunya cases registered annually in most municipalities and an increase in insecticide use for vector control. However, little was known previously about the insecticide resistance of mosquito populations, since only Araguaína and Palmas municipalities were monitored by MoReNAa Net and only for resistance to the organophosphate temephos.^7^ ^,^ ^21^ Comparing the data revealed, notably, that Araguaína maintained an average RR_95_ (8.8 in 2006, 12.9 in 2009, and 10.1 in the present study), while Palmas had an increase in RR_95_ (7.8 in 2006, 8.1 in 2009, and 13.1 in the present study).

The MoReNAa Net was dissolved in 2013 and the generation of data concerning insecticide resistance for *Aedes* populations stopped. In 2017, the Ministry of Health contacted two laboratories to start monitoring again, but out of the MoReNAa Net context.

Therefore, this study contributes substantially to our knowledge of the resistance profile via biological and molecular assays with different mosquito populations distributed all over the state. The generated data may help inform the vector control programs regarding the conscious use of insecticides as well as for decision making to achieve more effective, environmentally safe, and appropriate vector control for each individual site.
