# Supplementary material for: Evaluation of insecticide resistance in Aedes aegypti populations connected by roads and rivers: the case of Tocantins state in Brazil
Source: Mem Inst Oswaldo Cruz. 2019 Mar 25;114:e180318. doi: 10.1590/0074-02760180318 (PMC6434582; doi:10.1590/0074-02760180318)
Supplement: Supplementary file 1 [file 1678-8060-mioc-114-e180318-s.pdf]

TABLE

Probable number of dengue cases reported for the municipalities evaluated in this study, during the period from 2000-2012

Município	2000	2001	2002	2003	2004	2005	2006	2007	2008	2009	2010	2011	2012	Total
Araguaína	81	313	162	219	468	387	510	731	1.401	249	159	818	1.800	7.298
Araguatins	39	79	48	18	88	17	21	62	59	5	5	96	140	677
Caseara	0	2	0	0	0	0	3	3	9	4	1	7	5	34
Colinas do Tocantins	0	2	0	133	16	25	145	127	94	136	264	288	272	1.502
Guaraí	0	11	3	81	23	79	9	38	105	95	15	107	60	626
Gurupi	4	665	167	40	74	32	686	837	210	29	333	454	609	4.140
Palmas	828	1.438	603	673	16	1.482	1.404	5.283	2.971	1.454	2.728	5.743	4.923	29.546
Paraíso do Tocantins	36	765	125	36	40	148	895	378	493	73	602	561	362	4.514
Porto Nacional	8	79	19	10	2	11	231	380	171	273	841	152	121	2.298
Tocantinópolis	2	19	105	75	213	264	17	564	231	177	100	340	302	2.409

Source: Ministry of Health of Brazil. SINANonline/SVS/MS. Access in 06 Jul 2017.
